# Phenylethanol Glycosides from *Cistanche tubulosa* Suppress Hepatic Stellate Cell Activation and Block the Conduction of Signaling Pathways in TGF-β_1_/smad as Potential Anti-Hepatic Fibrosis Agents

**DOI:** 10.3390/molecules21010102

**Published:** 2016-01-18

**Authors:** Shu-Ping You, Long Ma, Jun Zhao, Shi-Lei Zhang, Tao Liu

**Affiliations:** 1 School of Public Health, Xinjiang Medical University, No. 393 Xinyi Road, Urumqi 830011, Xinjiang Uyghur Autonomous Region, China; youshupin@163.com (S.-P.Y.); malonglaoshi@163.com (L.M.); borlanzy@126.com (S.-L.Z.); 2 Key Laboratory for Uighur Medicine, Institute of Materia Medica of Xinjiang, No. 140 Xinhua South Road, Tianshan District, Urumqi 830000, Xinjiang Uyghur Autonomous Region, China

**Keywords:** *Cistanche tubulosa*, phenylethanol glycosides from *Cistanche* (CPHGs), echinacoside, acteoside, hepatic stellate cells (HSC), TGF-β1/smad

## Abstract

*Cistanche tubulosa* is a traditional Chinese herbal medicine widely used for regulating immunity and phenylethanol glycosides (CPhGs) are among the primary components responsible for this activity. Previous studies have indicated the preventive and therapeutic effects of CPhGs on bovine serum albumin (BSA)-induced hepatic fibrosis in rats. The aim of the study was to evaluate the anti-hepatic fibrosis effect of CPhGs and the monomers echinacoside and acteoside by inhibiting hepatic stellate cell (HSC) activation, blocking the conduction of signaling pathways in transforming growth factor-β_1_ (TGF-β_1_)/smad, and determine their *in vitro* hepatoprotective activity. HSC proliferation was obviously inhibited after treatment with CPhGs (100, 50 μg/mL)/echinacoside (500, 250, 125 μg/mL)/acteoside (6, 3 μg/mL), with IC_50_ values of 119.125, 520.345 and 6.999 μg/mL, respectively, in the MTT assay. Different concentrations of CPhGs/echinacoside/acteoside did not affect the cellular toxicity on HSC according to lactate dehydrogenase (LDH) measurements. Different concentrations of CPhGs/echinacoside/acteoside increased the mRNA level and protein expression of smad7, and decreased the mRNA levels of smad2, smad3 and the protein expression of smad2, phospho-smad2 (p-smad2), smad3, phospho-smad3 (p-smad3) in HSC. In summary, these results demonstrate that CPhGs/echinacoside/acteoside can block the conduction of the signaling pathways in TGF-β_1_/smad, and inhibit the activation of HSC, suggesting that *C. tubulosa* may thus be a potential herbal medicine for the treatment of liver fibrosis.

## 1. Introduction

The liver plays a central and crucial role in the regulation of toxic substance detoxification and synthesis of useful ones [1]. Hepatic fibrosis has been considered to be the wound-healing response of the liver to various toxic stimuli, including hepatitis, alcohol and immunostimulant compounds. Liver fibrosis is characterized by an excessive deposition of extracellular matrix (ECM) and activated hepatic stellate cells (HSCs) that can produce many cytokines and ECM in the liver, become proliferative and fibrogenic, subsequently accumulate ECM, and ultimately differentiate into fibrogenic myofibroblast-like cells. Transforming growth factor-β_1_ (TGF-β_1_) is regarded to be the main profibrogenic mediator. The signaling pathway related with TGF-β_1_ is an important mechanism of hepatic fibrosis, and it mainly includes smad-dependent and smad-independent signaling, and the smad-dependent signaling transduction pathway is thought to be a major channel of TGF-β_1_ signaling. Therefore, blockade of the TGF-β_1_/smad signaling pathway can suppress collagen production and eventually alleviate hepatic fibrosis, which has made this pathway an important target in anti-hepatic fibrosis drug research in recent years.

Despite the high worldwide incidence of hepatic fibrosis, no generally accepted anti-fibrogenic therapy is available [2,3,4]. Traditional Chinese Herbal Medicines (TCHMs) are of great interest for the treatment of liver disorders, because some can be used therapeutic drugs in the management and prevention of liver fibrosis. Presently, many studies are focused on potential anti-fibrogenic drugs that have been used as TCHMs for thousands of years [5].

**Figure 1:**
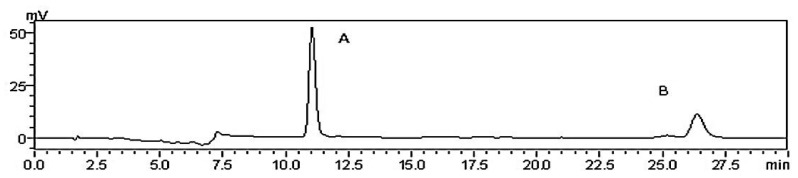
HPLC analysis of CPhGs. A, echinacoside; B, acteoside.

*Cistanche tubulosa W* (family Orobanchaceae), a parasitic plant, is widely grown in the southern region of Xinjiang in China [6]. People use it to invigorate the kidneys, nourish the blood, relax the bowels and delay senscence without side effects, and is officially listed in the Chinese Pharmacopoeia [7]. *C. tubulosa* contains a variety of active components, including phenylethanol glycosides (CPhGs), iridoids, and polysaccharides. Among the many active components in *C. tubulosa*, the CPhGs, which present a number of bioactivities (antioxidant, antifatigue, radioresistance, *etc.*) are the main characteristic components of this plant. In recent years, it is reported that CPhGs have excellent hepatoprotective effects, and can scavenge free radicals, protect hepatic membranes, and exhibit immunoregulatory effects, inhibition of apoptosis, inhibition of the expression of hepatitis B surface antigen (HBsAg) and hepatitis B e antigen (HBeAg) and hepatitis B virus (HBV) DNA replication activity, *etc.* HBV DNA level is the most reliable index that directly reflects the viral replication activity, which is the key factor in determining the natural history of chronic HBV infections, for monitoring the effect of antiviral therapy and assessing the prognosis of acute and chronic HBV infections. The serological detection indexes of HBV mainly include HBsAg, HBeAg, *etc.* [8,9,10,11]. However, there are few literature reports in the anti-hepatic fibrosis effects of CPhGs. Previous studies have indicated the preventative and therapeutic effects of CPhGs on bovine serum albumin (BSA)-induced hepatic fibrosis in rats, but the antifibrogenic activity of CPhGs and its major monomers (echinacoside and acteoside, Figure 1) have never been evaluated *in vitro*.

Therefore, the objective of this study was to evaluate the anti-hepatic fibrosis effect of CPhGs and the correpsnding monomers (echinacoside and acteoside) by inhibiting HSC activation, blocking the conduction of signaling pathways in TGF-β_1_/smad, and examine its *in vitro* hepatoprotective activity.

## 2. Results

### 2.1. Quantitative Determination of CPhGs

CPhGs in *C. tubulosa* contain two phenylethyl alcohol glycosides: echinacoside and acteoside, and their contents in the CPhGs were detected by HPLC analysis (Figure 1) to be 42.71% ± 0.42% and 14.27% ± 0.18%, respectively.

### 2.2. Inhibitory Activities of CPhGs, Echinacoside and Acteoside on HSC-T6

**Figure 2:**
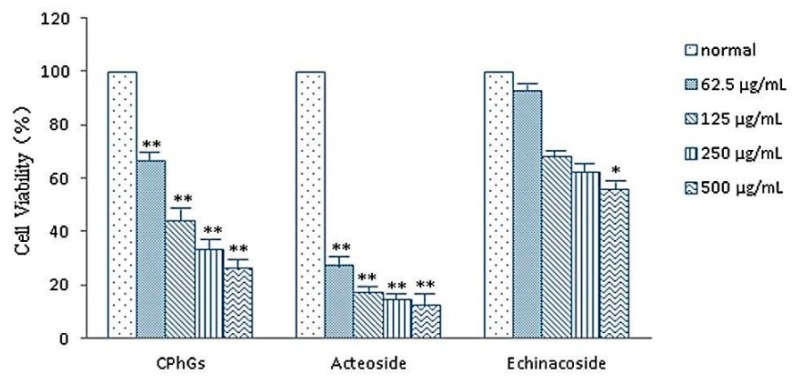
CPhGs, echinacoside and acteoside decreased the viability of HSC-T6. Cells were treated with DMEM (normal) or the indicated concentrations of CPhGs, echinacoside and acteoside for 48 h. Data were expressed as mean ± SD. *n* = 4. * *p* < 0.05, ** *p* < 0.01 compared with normal group.

CPhGs, echinacoside and acteoside (62.5–500 μg/mL) inhibited the cell viability of HSC in a concentration-dependent manner. CPhGs and acteoside showed a remarkable inhibitory activity on HSC cells, with 50% inhibition of cell growth activity (IC_50_) values was 119.125 μg/mL and 6.999 μg/mL respectively. Echinacoside displayed moderate inhibitory activity (IC_50_, 520.345 μg/mL; Table 1 and Figure 2).

**Table 1:** IC_50_ determination of CPhGs, echinacoside and acteoside on HSC.

Compound	Concentration (μg/mL)								
	500	250	125	62.5	31.25	15.625	7.8125	3.90625	Control
CPhGs OD	0.18	0.23	0.30	0.45	0.50	0.57	0.62	0.67	0.68
IR (%)	73.40	66.40	55.76	33.42	26.93	16.32	9.02	1.10	
Echinacoside OD	0.29	0.33	0.35	0.36	0.42	0.46	0.49	0.51	0.52
IR (%)	44.19	37.57	31.91	30.90	18.95	11.71	6.33	1.44	
acteoside OD	0.22	0.26	0.31	0.49	0.65	0.73	0.80	1.03	1.78
IR (%)	87.41	85.13	82.48	72.20	63.60	58.83	54.83	42.13	

OD is average absorbance ; IR is inhibition rate.

### 2.3. Cell Toxicity of CPhGs, Echinacoside and Acteoside on HSC

Lactate dehydrogenase (LDH), a stable cytoplasmic enzyme present in all cells, is rapidly released into the cell culture supernatant upon plasma membrane damage. Enzyme activity in the culture supernatant correlates with the proportion of lysed cells [12]. As shown in Table 2, when treated with different concentrations of CPhGs, echinacoside and acteoside, although LDH activity showed a slight increase compared to the cell control group, the the difference was not statistically significant (*p* > 0.05), indicating that most of the cell membranes maintained their integrity, and the inhibition of CPhGs, echinacoside and acteoside on HSC is not due to nonspecific cellular toxicity.

**Table 2:** Influence of CPhGs, echinacoside and acteoside on LDH secretion in HSCs.

Group	*n*	LDH (U/L)
Control	4	226.22 ± 26.82
CPhGs 100 μg/mL	4	240.58 ± 17.75 *
50 μg/mL	4	239.21 ± 2.11 *
25 μg/mL	4	230.50 ± 6.64 *
Echinacoside 500 μg/mL	4	240.01 ± 4.39 *
250 μg/mL	4	236.56 ± 13.06 *
125 μg/mL	4	233.02 ± 5.85 *
Acteoside 6 μg/mL	4	240.77 ± 6.43 *
3 μg/mL	4	240.13 ± 10.04 *
1.5 μg/mL	4	234.99 ± 7.92 *

Data are expressed as mean ± SD. * *p* > 0.05, compared with normal group, there was no significant difference.

### 2.4. Effect of CPhGs, Echinacoside and Acteoside on HSC Proliferation Induced by TGF-β_1_

The proliferation, activation and differentiation of HSC are regulated by a variety of factors. Among the various factors involved in liver fibrosis, TGF-β_1_ is considered to be the most important, as it can activate HSC, promote myofibroblast differentiation, increase ECM synthesis, and inhibit ECM degradation, and release chemokines and cytokines involved in liver fibrosis [13]. In this study, except for the control group, stationary HSCs were stimulated with TGF-β_1_ *in vitro* to simulate the formation of early liver fibrosis, and to investigate the effect of CPhGs, echinacoside and acteoside on TGF-β_1_-induced HSC proliferation. As shown Table 3, compared with control group, the TGF-β_1_ group significantly promoted the proliferation of HSC (*p* < 0.01). Compared with the TGF-β_1_ group, TGF-β_1_ + CPhGs (TC) (50, 100 μg/mL, *p* < 0.05), TGF-β_1_ + echinacoside (TE) (125, 250, 500 μg/mL, *p* < 0.05), TGF-β_1_ + acteoside (TA) (3.0, 6.0 μg/mL, *p* < 0.05) all remarkably inhibited the proliferation of HSCs induced by TGF-β_1_ to different degrees.

**Table 3:** Inhibition effects of CPhGs, echinacoside and acteoside with different doses on TGF-β_1_-induced HSC proliferation.

Group	Absorbance Inhibition (OD) (%)		Group	Absorbance Inhibition (OD) (%)		Group	Absorbance Inhibition (OD) (%)	
Control TGF-β_1_	0.91 ± 0.05 1.30 ± 0.05 **		Control TGF-β_1_	0.53 ± 0.06 0.74 ± 0.05 **		Control TGF-β_1_	0.80 ± 0.07 0.96 ± 0.03 **	
TC 100 μg/mL	1.14 ± 0.14 ^#^	41.60	TE 500 μg/mL	0.61 ± 0.01 ^##^	55.58	TA 6.0 μg/mL	0.87 ± 0.03 ^##^	53.44
TC 50 μg/mL	1.15 ± 0.05 ^#^	38.40	TE 250 μg/mL	0.67 ± 0.03 ^#^	30.89	TA 3.0 μg/mL	0.89 ± 0.04 ^#^	45.50
TC 25 μg/mL	1.22 ± 0.08	19.68	TE 125 μg/mL	0.68 ± 0.02 ^#^	25.83	TA 1.5 μg/mL	0.93 ± 0.04	20.92

Data are expressed as mean ± SD. ** *p* < 0.01 compared with normal group, ^#^ *p* < 0.05, ^##^ *p* < 0.01 compared with the model group.

### 2.5. Expressions of smad2, smad3 and smad7 mRNA after CPhGs, echinacoside and acteoside intervention on HSCs

**Figure 3:**
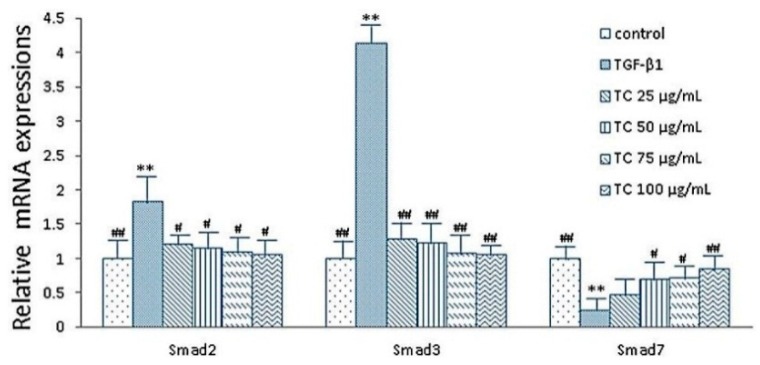
Effects of CPhGs on the expressions of smad2, smad3 and smad7 in HSC (RT-PCR assay). Data were expressed as mean ± SD. ** *p* < 0.01 compared with control group, ^#^ *p* < 0.05, ^##^ *p* < 0.01 compared with TGF-β_1_ group.

Smad3 and smad2 are key downstream effectors of the TGF-β signaling pathway [14,15], and smad7 is an inhibitor of smad signaling. Theoretically, the increased expression of smad7 or decreased expression of smad2/smad3 can block the conduction of signaling pathways in TGF-β_1_/ smad. As shown in Figure 3, real-time quantitative PCR analysis showed that the mRNA expression of smad2 and smad3 indicated a lower level, while smad7 indicated higher levels in the control group, compared with the TGF-β_1_ group. In the TC (25, 50, 75, 100 μg/mL) group, the mRNA level of smad2 (*p* < 0.05) and smad3 (*p* < 0.01) were significantly decreased compared with the TGF-β_1_ group, and was even similar to the control group; meanwhile, the mRNA level of smad7 was significantly increased in the TC (50, 75, 100 μg/mL) group (*p* < 0.05, *p* < 0.05, *p* < 0.01, respectively). However, the smad7 expression indicated a ascending trend in the TC 25 μg/mL group compared with the TGF-β_1_ group (Figure 3).

**Figure 4:**
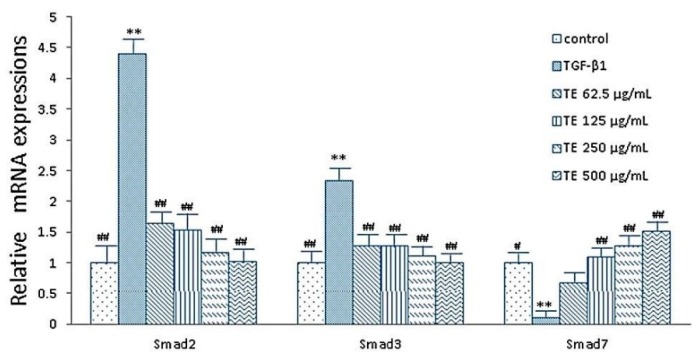
Effects of echinacoside on the expressions of smad2, smad3 and smad7 in HSC (RT-PCR assay). Data were expressed as mean ± SD. ** *p* < 0.01 compared with control group, ^#^ *p* < 0.05, ^##^ *p* < 0.01 compared with TGF-β_1_ group.

**Figure 5:**
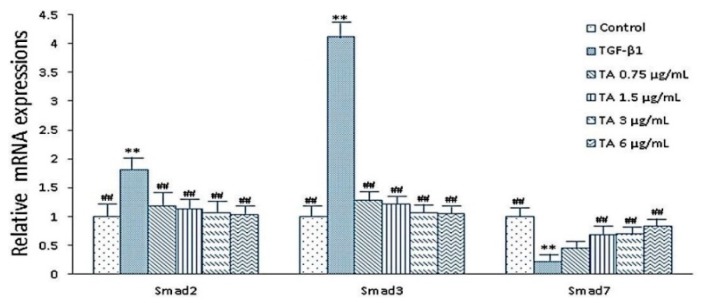
Effects of acteoside on the expressions of smad2, smad3 and smad7 in HSC (RT-PCR assay). Data were expressed as mean ± SD. ** *p* < 0.01 compared with control group, ^##^ *p* < 0.01 compared with TGF-β_1_ group.

Meanwhile, compared with the TGF-β_1_ group, expression of smad2 and smad3 mRNA were significantly decreased in the TE (62.5, 125, 250, 500 μg/mL) group (*p* < 0.01), and expressions of smad7 mRNA were significantly increased in the TE (125, 250, 500 μg/mL) group (*p* < 0.01) (Figure 4). Similarly, TA (0.75, 1.5, 3.0, 6.0 μg/mL) group also showed the same trend (Figure 5).

### 2.6. Influence of CPhGs, Echinacoside and Acteoside on TGF-β_1_/smad Signaling Pathway in HSC

**Figure 6:**
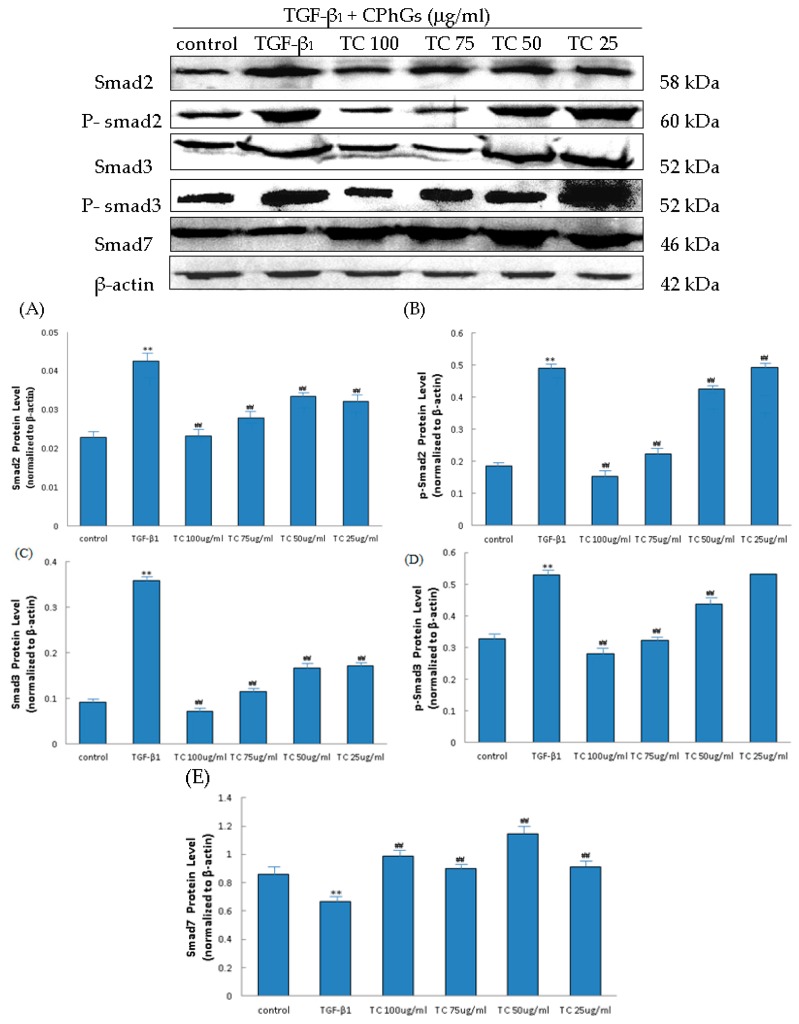
Effects of CPhGs on the protein expression of smad2, p-smad2, smad3, p-smad3 and smad7 in HSC. (**A**) Smad2 protein level; (**B**) P-smad2 protein level; (**C**) Smad3 protein level; (**D**) P-smad3 protein level; (**E**) Smad7 protein level. Data are expressed as mean ± SD. ** *p* < 0.01 compared with control group, ^##^ *p* < 0.01 compared with TGF-β_1_ group.

**Figure 7:**
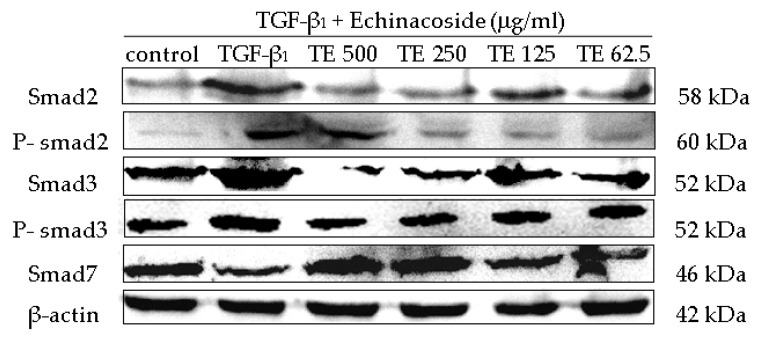

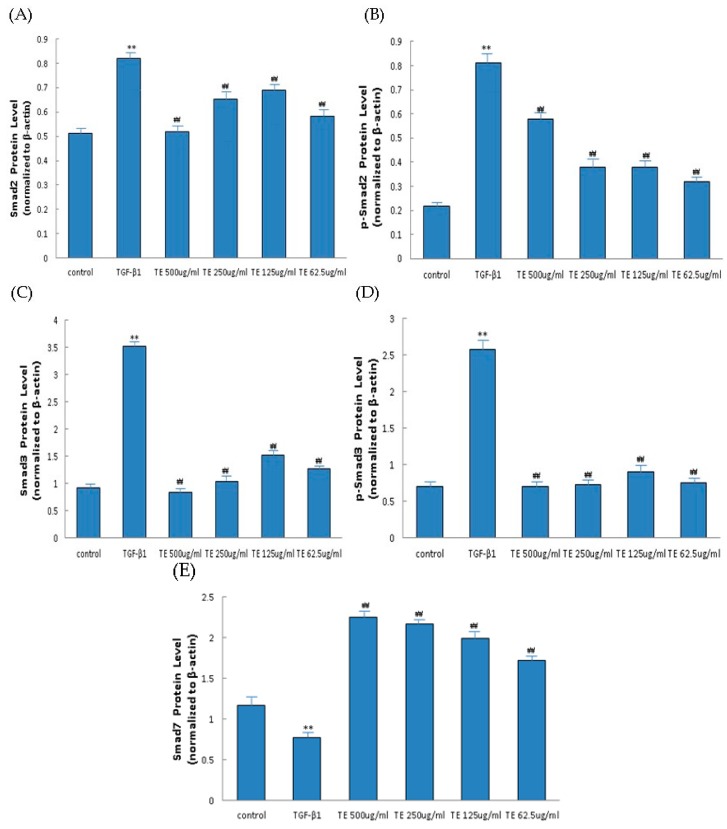
Effects of echinacoside on the protein expression of smad2, p-smad2, smad3, p-smad3 and smad7 in HSC. (**A**) Smad2 protein level; (**B**) P-smad2 protein level; (**C**) Smad3 protein level; (**D**) P-smad3 protein level; (**E**) Smad7 protein level. Data were expressed as mean ± SD. ** *p* < 0.01 compared with control group, ^##^ *p* < 0.01 compared with TGF-β_1_ group.

**Figure 8:**
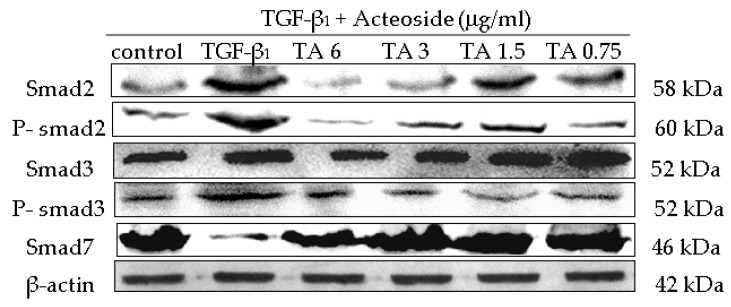

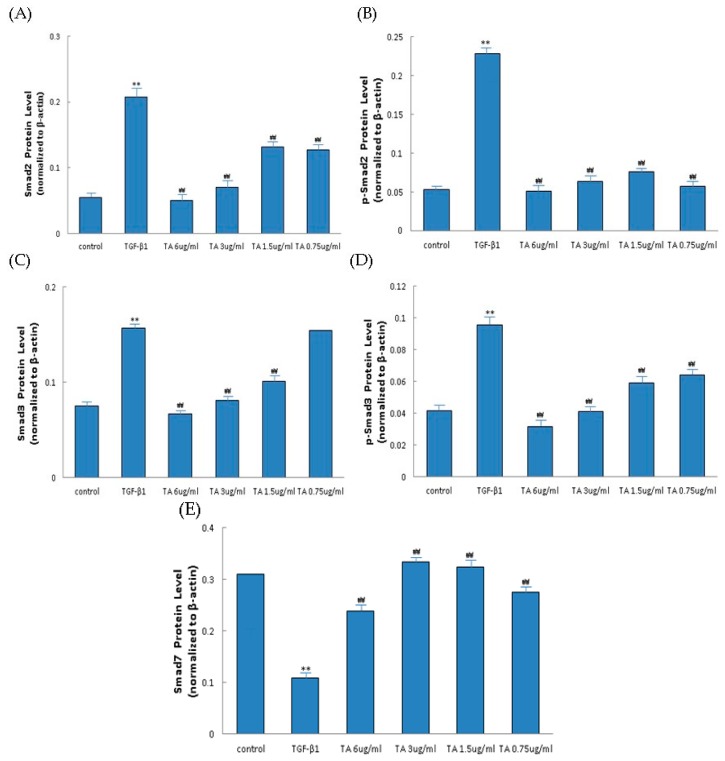
Effects of acteoside on the protein expression of smad2, p-smad2, smad3, p-smad3 and smad7 in HSC. (**A**) Smad2 protein level; (**B**) P-smad2 protein level; (**C**) Smad3 protein level; (**D**) P-smad3 protein level; (**E**) Smad7 protein level. Data were expressed as mean ± SD. ** *p* < 0.01 compared with control group, ^##^ *p* < 0.01 compared with TGF-β_1_ group.

The TGF-β_1_/smad signaling pathway depends on smad2, smad3, phospho-smad2 (p-smad2), phospho-smad3 (p-smad3) and smad7, where p-smad2, p-smad3 are the activated forms of smad2 and smad3. In this study, the protein levels of smad2, smad3, p-smad2, p-smad3 and smad7 were analyzed. Western blot analysis detected increases in the smad2, smad3, p-smad2, p-smad3 levels by TGF-β_1_, and the inhibition of these increases by CPhGs, echinacoside and acteoside; meanwhile, western blot analysis revealed upregulated smad7 levels by CPhGs, echinacoside and acteoside. (Figure 6, Figure 7 and Figure 8).

As shown in Figure 6, compared with the control group, the protein expressions of smad2, smad3, p-smad2, p-smad3 were significantly increased, and smad7 protein expression was decreased in the TGF-β_1_ group. In the TC (25, 50, 75, 100 μg/mL) drug groups, the smad2 (Figure 6A), p-smad2 (Figure 6B), smad3 (Figure 6C), p-smad3 (Figure 6D) expressions were significantly lower than that in the TGF-β_1_ group (*p* < 0.01), and smad7 (Figure 6E) protein expression was higher than that of the TGF-β_1_ group (*p* < 0.01) (Figure 6).

At the same time, the expressions of smad2, smad3, p-smad2, p-smad3 and smad7 protein were measured after echinacoside and acteoside intervention on HSC. As shown in Figure 7 and Figure 8, the smad2, smad3, p-smad2 and p-smad3 protein expression levels were significantly inhibited (*p* < 0.05 or *p* < 0.01), smad7 protein expression level was elevated (*p* < 0.01), compared with the TGF-β_1_ group (Figure 7 and Figure 8).

## 3. Discussion

Liver fibrosis is one of the leading causes of morbidity and mortality worldwide, but very limited therapeutic options are currently available for this condition, therefore, it is very important for us to seek potential targets for therapeutic intervention to prevent the development of liver fibrosis [16]. Recently, researchers have found that various herbal medicines have hepatoprotective or anti-fibrotic effects. Many TCHMs such as Fufang Biejia Ruangan pills [17] and Xiao-chai-hu decoction (shosaiko to in Japan) [18,19] have been widely used to treat hepatic fibrosis in China, Korea, and Japan for thousands of years. *C. tubulosa* contains a variety of active components including CPhGs, iridoids, and polysaccharides. As one of efficaceous material basis of *C. tubulosa*, CPhGs possess excellent hepatoprotective activity, but there is limited information about the anti-fibrotic effects of GPhCs and its related compounds. In this study, we investigated how CPhGs, echinacoside and acteoside suppress HSC activation and block the signaling pathway conduction in TGF-β_1_/smad as potential anti-hepatic fibrosis agents *in vitro*.

Liver injury of any etiology will ultimately lead to activation of HSCs, which undergo transdifferentiation to fibrogenic myofibroblast-like cells [20]. That is to say that myofibroblasts are derived from the activation and proliferation of HSCss [21], and it is regarded as the mediator of hepatic fibrosis formation. For this reason, we performed *in vitro* studies to compare the cell viability rates of HSCs exposed to CPhGs, echinacoside and acteoside. The results showed that inhibitory effect of acteoside on HSC was the strongest, and the effect of CPhGs was better than that of echinacoside. CPhGs, echinacoside and acteoside medicated sera all inhibited HSC proliferation in a dose-dependent manner.

LDH is a cytoplasmic enzyme in living cells, and it cannot penetrate the cell membrane under normal circumstances. When cells are damaged, the membrane permeability increases, and LDH is released to cell exterior. Usually, LDH can detect the degree of cell damage [22,23,24]. The investigation showed that the LDH activity of CPhGs, echinacoside and acteoside medicated serum only showed a slight increase compared with the cell control group, and the difference was not statistically significant (*p* > 0.05), indicating that most of the cell membrane maintained their integrity, and inhibition of HSCs by CPhGs, echinacoside and acteoside is not due to nonspecific cellular toxicity.

When liver injury occurs, activated hepatocytes can release TGF-β_1_ that in turn activates HSC-T6, therefore, TGF-β_1_ has a close relationship with fibrosis [25,26,27]. In this study, CPhGs, echinacoside and acteoside could be considered as an attractive therapeutic strategy for inhibition of HSC proliferation after HSCs were stimulated with wTGF-β_1_ *in vitro*. We speculate that CPhGs, echinacoside and acteoside may be used as a kind of treatment and/or prevention of liver fibrosis, and inhibit the formation of early liver fibrosis. The formation of hepatic fibrosis is a complex process of multifactor and multicell involvement. In this pathological process, the cell-cell, cell-cytokines and cell-matrix interactions constitute a cumbersome network. In this network, the development of hepatic fibrosis can be regulated by different signaling pathways and means. TGF-β_1_ is a classic activator of HSC and a key mediator in the pathogenesis of liver fibrosis [28]. CPhGs, echinacoside and acteoside can inhibit the expressions of TGF-β_1_/smad pathway-related mRNA and proteins in HSCs.

TGF-β_1_ exerts its cellular effects via the smad signaling pathway, and it has been considered a key mediator in the development of liver fibrosis and inflammation. Upon binding of TGF-β_1_ to the TGF-β type II receptor, the type II receptor kinase phosphorylates the *GS* domain of TGF-β type I receptor, leading to the activation of type I receptor [29]. Via the action of p-smad2 and p-smad3 at two serine residues in the SSXS motif of their C terminal, reactions downstream of the smad signaling pathway are activated by the activation of type I receptor [30]. P-smad2 and p-smad3 form oligomeric complexes with smad4, then enter the nucleus and exert their biological transcription activity. Thus, smad proteins transmit signals directly from the receptor kinase to the nucleus [31]. On the other hand, smad7 is firmly combined with TGF-β1 receptor, leading to the inability of smad 2/3 to be activated as well as inhibition of the signal transduction pathways [32]. Smad7 can act to inhibit the p-smad2 and p-smad3, nuclear translocation of activated smad complexes, and activation of (CAGA) (9)-MLP-Luc, resulting in decreased collagen I expression and complete inhibition of TGF-β signal transduction [33,34]. Our results showed that CPhGs, echinacoside and acteoside can decrease smad2 and smad3 mRNA expressions, and increase smad7 mRNA expression; inhibit smad2, p-smad2, smad3 and p-smad3 protein expressions, and up-regulate smad7 protein expression, suggesting that CPhGs, echinacoside and acteoside also plays a role in inhibiting HSC activation and thus inhibit hepatic fibrosis, highlighting a potential anti-fibrotic mechanism.

The sequence of the hepatoprotective effects of the three testing agents was shown to be acteoside > CPhGs > echinacoside. In order to further elucidate the reasons for the differences in the mechanism by which CPhGs, echinacoside and acteoside protect against liver fibrosis, their chemical structures were analyzed. The phenethyl alcohol glycoside moiety seem to be an essential structure for the hepatoprotective effect [35]. Acteoside (C_29_H_36_O_15_) is a double glycoside, and echinacoside (C_35_H_46_O_20_) is a triglycoside, *i.e.*, echinacoside has an extra glycoside in its structure compared to acteoside, which leads to an increase in its spatial size. The hepatoprotective or inhibition of HSC activity of acteoside is better than that of echinacoside, which may be because of the existence of steric hindrance.

## 4. Experimental Section

### 4.1. Chemicals and Reagents

TGF-β_1_ was purchased from Peprotech (Rocky Hill, NJ, USA). The HSC-T6 cell line was purchased from Wuhan Procell Gene Bio-technology Co., Ltd. (Wuhan, China). Dimethyl sulfoxide (DMSO) was purchased from Sigma Inc. (St. Louis, MO, USA). Smad2, smad3 and smad7 primers were produced by Shanghai Sangon Biological and Technological Company (Shanghai, China). The Revert Aid first strand cDNA synthesis kit was purchased from Thermo Scientific (Shanghai, China). The Quantifast SYBR green PCR kit was purchased from QIAGEN GmbH (Hilden, Germany). p-smad2 (ser465/467) antibody, Smad3 (C67H9) rabbit mAb and p-smad3 (ser423/425) rabbit mAb were purchased from Cell Signaling Technology (Boston, MA, USA). Smad7 antibody was purchased from Boster (Wuhan, China). Smad2 polyclonal antibody and β-actin monoclonal antibody were purchased from Proteintech (Wuhan, China). Detection of primary antibody was done using 2° Antibody solution (Alk-Phos. Conjugated, Anti-rabbit) and 2° Antibody solution (Alk-Phos. Conjugated, Anti-mouse) purchased from Invitrogen™ (Carlsbad, CA, USA). Lactate dehydrogenase assay kit was from Nanjing Jiancheng Bioengineering Institute (Nanjing, China).

### 4.2. Plant Material

*C. tubulosa* was collected from Tulufan, in the Xinjiang Uirghur autonomous region of China, in May 2006. The plant materials were identified as *C. tubulosa* by Jiang He, Institute of Materia Medica of Xinjiang. A voucher specimen was deposited at the Institute of Materia Medica of Xinjiang in China.

### 4.3. Isolation and Purification of CPhGs and Its Compounds

**Figure 9:**
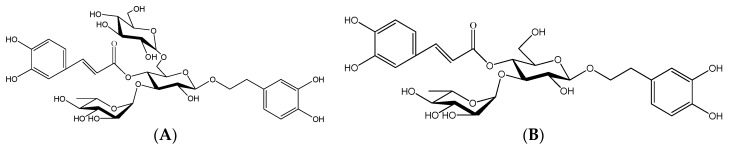
Structures of echinacoside (**A**) and acteoside (**B**).

Dried and sliced rhizomes of *C. tubulosa* (6.0 kg) were consecutively extracted three times under reflux with 70% ethanol (4.8 L), and the solvent was then removed from the combined extracts to to yield the ethanol extract (1.146 kg). The ethanol extracts were purified by AB-8 resin to obtain the crude phenyl ethanol glycosides (CPhGs) extract. After dissolving in water, the extract was purified by AB-8 adsorption macroporous resin chromatography to obtain the total phenyl ethanol glycosides from *C. tubulosa* (CPhGs, 210 g). CPhGs were applied on an ODS RP-18 column and eluted successively with mixtures of MeOH–H_2_O (0:1 → 1:0). Eluates were combined into five subfractions according to TLC behavior using the solvent system CHCl_3_-MeOH-H_2_O (6:4:0.5). Spots were visualized after spraying with 1% FeCl_3_.Various fractions were repeatedly purified by Sephadex LH-20 column chromatography with methanol as eluent, and two phenyl ethanol glycosides were isolated from the CPhGs. Their structures were confirmed as echinacoside (C_35_H_46_O_20_) and acteoside (C_29_H_36_O_15_) using MS, ^1^H-, and ^13^C-NMR [35], which purities were determined to be 99.17% and 98.92%, respectively, by UV-HPLC. The structures of echinacoside and acteoside are shown in Figure 9.

### 4.4. Quantitative Determination of CPhGs

Liquid chromatography (HPLC) (LC-10A HPLC instrument, Shimadzu Co., Kyoto, Japan) was employed to analyze the contents of echinacoside and acteoside in the CPhGs. A Phenomenex Gemini ODS column (250 × 4.6 mm, 5 µm) was used at 30 °C. An isocratic mobile phase consisting of methanol–acetonitrile–1% acetic acid (15:10:75, *v*/*v*/*v*) was used for a 40 min run, at a flow rate of 0.6 mL/min, with UV detection at 334 nm.

### 4.5. Cell Culture

HSC cells were cultured in Dulbecco’s modified Eagle’s medium (High Glucose) (DMEM, Beijing, China) supplemented with 10% fetal bovine serum (FBS, Gibco, Carlsbad, CA, USA), 100 IU/mL penicillin and 100 μg/mL streptomycin (HyClone, Logan, UT, USA) in a humidified incubator at 37 °C with 5% CO_2_. These cells were regularly subcultured by trypsin 0.25% (1×) solution (HyClone, Logan, UT, USA) and the medium was changed every 2 days. Initially, cells were cultured with DMEM containing 10% FBS for 48 h. The medium was then replaced with DMEM without FBS to starve the cells for 12 h. These cells were propagated for 48 h and After 48 h, serum was starved with 0.5% FBS for 12 h before adding 5 ng/mL TGF-β_1_.

### 4.6. Determination of IC_50_ Values

After an overnight incubation in starvation medium containing 0.5% FBS, HSC cells were seeded in a 96-well plate at a density of 5 × 104 cells/mL for 24 h. HSC cells were treated with CPhGs, echinacoside and acteoside at different concentrations (0, 3.90625, 7.8125, 15.625, 31.25, 62.5, 125, 250, and 500 μg/mL) for 48 h. Each concentration had four wells. At the end of the treatment, 20 μL MTT [3-(4,5-dimethylthiazol-2-yl)-2,5-diphenyltetrazolium bromide] (Sigma; 5 mg/mL) was added and the cells were incubated for another 4 h. DMSO (200 μL) was added to each well after removing the supernatant. After shaking the plate for 10 min on a shaker, the IC_50_ values were obtained by measuring the absorbance at 490 nm wavelength using an enzyme-labeling instrument (Benchmark PLUS, Hercules, CA, USA), this assay was done triplicate. The IC_50_ values of CPhGs, echinacoside and acteoside were 119.125, 520.345 and 6.999 μg/mL, respectively.

### 4.7. The Cell Proliferative Inhibition Effects and Cell Viability

HSC cells were exposed to different concentrations of CPhGs (25, 50, 100 μg/mL), echinacoside (125, 250, 500 μg/mL) and acteoside (1.5, 3, 6 μg/mL). Different concentrations of CPhGs, echinacoside and acteoside were applied on the plate in four consecutive wells and incubated for 48 h. The cell proliferation inhibition effects and the level of cell viability (based on the measurement of LDH activity released from damaged cells into the supernatant [36]) were determined by an MTT assay. The inhibition rate was calculated using the following formula [37]:

Inhibition rate (%) = [1 − (average absorbance of experimental group/average absorbance of blank control group)] × 100%
(1)

According to the kit’s instruction,

LDH (U/L) = (Measured OD − control OD)/(standard OD − blank OD) × 0.2 mmol/L × 1000
(2)

Our preliminary study showed that CPhGs, echinacoside and acteoside did not affect the cell viability.

### 4.8. Anti-Proliferative Activities by TGF-β_1_

HSC activated by TGF-β_1_ has been long considered to be associated with liver fibrosis, and inhibition for HSC growth has been proposed as a method for treating liver fibrosis [36,38]. The anti-proliferative effects of CPhGs, echinacoside and acteoside on HSCs activated by TGF-β_1_ were determined by an MTT assay [39]. Using the procedures and drug concentrations as described, the experimental groups included the control group, TGF-β_1_ group, TGF-β_1_ + different concentration drug groups. All cell groups except the control group were cultured with DMEM containing 5.0 ng/mL TGF-β_1_ (without FBS) for 24 h. Inhibitory activity on cell proliferation was calculated as 100 × (absorbance of treated compound − absorbance of background light)/(absorbance of model − absorbance of background light).

### 4.9. Reverse Transcription-Polymerase Chain Reaction (RT-PCR)

The mRNA expression level of smad2, smad3 and smad7 were determined by real-time PCR. To determine mRNA expression in HSC cells, the cells (5 × 10^4^ cells) were seeded in six-well plates with 3.0 mL DMEM with 10% FBS and incubated overnight at 37 °C and 5% CO_2_. After culture media was changed to serum-free DMEM, CPhGs (100, 50, 25 μg/mL), echinacoside (500, 250, 125 μg/mL) and acteoside (6, 3, 1.5 μg/mL) were added to the wells. After incubation for 48 h with CPhGs and monomer compositions, total RNA was extracted using TRIzol reagent (Invitrogen, Carlsbad, CA, USA) and agitated vigorously with chloroform for 15 s. After allowing to stand at room temperature for 3 min, the lysate was centrifuged at 12,000× *g* for 15 min at 4 °C. RNA in the aqueous phase was precipitated with isopropanol, and the upper aqueous phase was transferred to a new microcentrifuge tube. RNA was precipitated by adding 0.75% ethanol, after which the microcentrifuge tube and centrifuged at 12,000× *g* at 4 °C for no more than 5 min. The supernatant was removed and the RNA was dried at room temperature for 5–10 min. The primers (Sangon) were designed using Batch Primer 3, and are listed in Table 4. The results were normalized to the mRNA of the housekeeping gene β-actin as an internal control and are presented as relative mRNA levels. Reactions were performed with 8 μL iQ SYBR Green Supermix, 1 μL 10 pM primer pair, 8.5 μL distilled water, and 2.5 μL cDNA. Each polymerase chain reaction was performed under the following conditions: 95 °C for 3 min, then 40 cycles of 10 s at 95 °C, 30 s at 55 °C, and 10 s at 55 °C–95 °C for extension, followed by a single fluorescence measurement. The final results were described with the relative values (2^−ΔΔCt^). The calculation and analysis were performed by iQ5 Real Time PCR Detection System.

**Table 4:** Primer sequences for RT-PCR.

Target Gene Primer Sequence (Forward/Reverse) (Rat)			GenBank
Smad2	F: ACGGCTTTACAGATCCATCG	R: GCCAGAAGAGCAGCAAATTC	NM_0012774501
Smad3	F: GGCCATGTTGGTTTATGGAG	R: CCAGGGTGAAGATGACAGGT	NM_013095.3
Smad7	F: GTGGCATACTGGGAGGAGAA	R: TGGAGAAACCAGGGAACACT	NM_030858.1
β-actin	F: TAAGGCCAACCGTGAAAAGATG	R: AGAGGCATACAGGGACAACACA	NM_031144.3

### 4.10. Western Blot Analysis

Whole cell extracts were prepared using Radioimmunoprecipitation Assay (RIPA) buffer (Thermo Fisher Scientific, Inc.) with 1% Halt protease inhibitor and 1% Halt phosphatase inhibitor cocktails (Thermo Fisher Scientific, Inc.). The total protein was used for the detection of smad2, p-smad2, smad3, p-smad3 and smad7. The protein concentration was measured and quantified by the Bradford method. Protein (10–30 μg) was separated on a 10% SDS-PAGE gel and transferred to polyvinylidene fluoride (PVDF) membranes (Millipore, Boston, MA, USA). Membranes were blocked for 1 h at room temperature with 5% bovine serum albumin, and the primary antibodies (smad2 polyclonal antibody, p-smad2 antibody, smad3 rabbit mAb and p-smad3 rabbit mAb, 1:1000 dilution; rabbit mAb of smad7, 1:200 dilution, β-actin monoclonal antibody, 1:5000 dilution) were incubated at 4 °C overnight. The corresponding Alk-Phos. conjugated secondary antibodies were incubated at room temperature. Finally, the membranes were washed three times with 1 x Tris-HCl saline with 0.1% Tween 20, and signals were scanned and visualized by a GEL DOC XR Imaging System (Bio-Rad, Hercules, CA, USA). Densitometric analysis was performed on the proteins of interest and normalized to β-actin by GEL DOC Image Studio software (Bio-Rad). β-actin was used as the internal control.

### 4.11. Statistical Analysis

Data were expressed as means ± standard deviation (SD). Statistical analyses were performed using the SPSS 16.0 software (Xinjiang Medical University). Probit analysis was used to analyze IC_50_ values. The significance of difference was calculated by one-way ANOVA test, and values with *p* < 0.05 were considered to be statistically significant.

## 5. Conclusions

In conclusion, CPhGs from *C. tubulosa* and its components echinacoside and acteoside show significant anti-fibrotic effects. Among them, the activity of acteoside is the most powerful, while the activity of the CPhGs is between the two compounds. Inhibition of activation of TGF-β_1_/Smad signaling may be the underlying mechanism by which CPhGs protect against chronic liver disease associated with fibrosis, and echinacoside and acteoside are the effective anti-fibrotic material basis of *C. tubulosa*.

## Abbreviations

The following abbreviations are used in this manuscript:

CPhGs	Phenylethanol glycosides from cistanche
BSA	Bovine serum albumin
HSC	Hepatic stellate cells
mAb	Monoclonal antibody
TGF-β1	Transforming growth factor-β1
IC50	Inhibition of 50% of cell growth activity
ECM	Extracellular matrix
TCHM	Chinese and herb medicine
TC	TGF-β1 + CPhGs
TE	TGF-β1 + echinacoside
TA	TGF-β1 + acteoside
p-smad2	phosphorylation of smad2
p-smad3	phosphorylation of smad3
LDH	Lactate dehydrogenase
HPLC	Liquid chromatography
RIPA	Radioimmunoprecipitation assay
RT-PCR	Reverse transcriptase polymerase chain reaction
SDS-PAGE	Sodium dodecyl sulfate polyacrylamide gel electrophoresis
